# Parathyroid autotransplantation in extensive head and neck resections: case series report

**DOI:** 10.1186/1477-7819-9-149

**Published:** 2011-11-15

**Authors:** Panagiotis G Athanasopoulos, Maria Kyriazi, Nikolaos Arkadopoulos, Dionysios Dellaportas, Asimina Manta, Theodosios Theodosopoulos, Aliki Tympa, Ioannis Vassileiou, Vassilios Smyrniotis

**Affiliations:** 1 2nd Department of Surgery, Aretaieion Hospital, University of Athens Medical School, 1st Rimini Street, Chaidari 12462, Greece; 2 4th Department of Surgery, Attikon Hospital, University of Athens Medical School, 1st Rimini Street, Chaidari 12462, Greece; 3 1st Department of Anaesthesiology, Aretaieion Hospital, University of Athens Medical School, 76 Vasilissis Sofias Avenue, Athens 11528, Greece; 4 Puddicombe Way, Milton House (Flat 11B), Cambridge CB2 0AD, UK

**Keywords:** parathyroid autotransplantation, transhiatal oesophagolaryngopharyngeal resection, extensive oesophageal resection, postoperative hypoparathyroidism

## Abstract

Permanent or temporary hypoparathyroidism may be a debilitating result of radical cervical surgery, as noted most commonly following thyroid or parathyroid surgery. However, it can also be the outcome of any surgical procedure involving bilateral extensive manipulation of the anterior neck triangle, especially in order to ensure oncologically adequate surgical margins.

We report our experience of three patients that underwent parathyroid immediate autotransplanation following extensive surgical manipulations of the neck region for oncological reasons. PTH levels were restored to normal by the fourth postoperative week, allowing us to wean the patients off calcium and vitamin D_3 _supplementation, which was attributed to full autograft function. Parathyroid autotransplantation, immediate or delayed, is a simple and safe technique which should be considered by the surgeon whenever there is a high risk for postoperative hypoparathyroidism following radical operations of the neck for oncological reasons.

## Background

Permanent or temporary hypoparathyroidism may be a debilitating result of radical cervical surgery, as noted most commonly following thyroid or parathyroid surgery. However, it can also be the outcome of any surgical procedure involving bilateral extensive manipulation of the anterior neck triangle [1-3]. Oesophageal carcinomas in the proximity of the cricopharyngeal junction in particular, may require the en bloc resection of adjacent organs, such as the larynx, the thyroid gland and the parathyroids, as well as a complete neck dissection in order to achieve an oncologically satisfactory outcome. Severe postoperative hypoparathyroidism becomes clinically evident with symptoms including paresthesias, muscle cramping, shortness of breath secondary to bronchospasm, tetanic contractions, distal extremity numbness, seizures and QT prolongation in the electrocardiogram. In order to deal with these agonizing for the patient and clinician symptoms long-term medication supplementation, frequent laboratory testing and repeated hospital admissions may be required [4]. Furthermore, the lack of established parathormone replacement medical therapy to date results in severely afflicting the patient's quality of life postoperatively.

Parathyroid autotransplantation has long been employed as a means of preventing permanent hypoparathyroidism after thyroid or parathyroid surgery [5]. However, it is not usually considered as an option in extensive head and neck dissections performed for other causes, although the risk of inadvertent damage to the parathyroids still exists. We report our experience of three patients in which parathyroid autotransplantation was utilized to avoid postoperative hypoparathyroidism following oesophagectomy combined with resection of adjacent structures.

## Case Series Presentation

The first patient was a 65-year-old female, suffering from an oesophageal carcinoma of the cricopharyngeal junction, who underwent a transhiatal oesophagolaryngopharyngeal resection with bilateral neck dissection. The second patient, a 70-year-old female, also underwent transhiatal oesophagolaryngopharyngeal resection and bilateral neck dissection for a hypopharyngeal carcinoma. Lastly, the third patient was a 63-year-old male who was subjected to transhiatal oesophagectomy and total thyroidectomy for a carcinoma of the lower third of the oesophagus and a concomitant toxic goiter. Permanent tracheostomy was considered mandatory in patients 1 and 2, as their tumour directly invaded larynx. In the 3^rd ^patient transhiatal oesophagectomy was combined with total thyroidectomy for a toxic goiter, which was firmly attached to the oesophagotracheal groove rendering the patient vulnerable to hypoparathyroidism. The continuity of the alimentary tract was restored by right colon interposition and anastomosis to the pharynx in patient 1, whereas in patient 2 and 3 an immediate cervical pharyngogastric and oesophagogastric anastomosis was performed respectively.

Taking into account the extensive neck dissection required for these surgical procedures, all patients were deemed high risk for inadvertent damage to the parathyroid glands and the subsequent development of hypoparathyroidism postoperatively. Therefore, it was decided that they should be submitted to parathyroid autotransplantation. During surgery the two upper parathyroid glands were identified, resected and stored in sterile iced saline until completion of the operation. Pathological examination confirmed their accurate identification and multiple frozen sections of the surrounding fatty tissue precluded the presence of malignancy. Then, the two parathyroids were sliced in pieces of 1 × 3 mm in diameter, and three to four grafts were implanted on separate muscle pockets in the sternocleidomastoid muscle or, in the 3^rd ^case, in the non-dominant forearm. A total of 20 pieces were grafted in every patient. Each muscle pocket was re-approximated using non-absorbable sutures.

Pathological examination confirmed that at least 2 parathyroids had been excised en bloc with the resected surgical specimen (the two inferior glands were identified in cases 1 and 3, and three glands in case 2).

**Figure 1:**
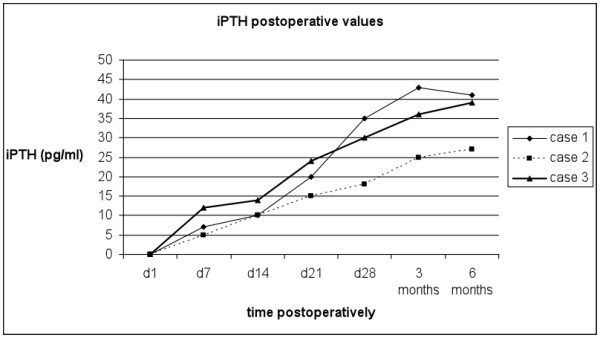
**Postoperative intact PTH levels of the three patients**. Postoperative intact PTH levels of the three patients (normal range 15-65 pg/ml, 2^nd ^generation PTH Cobas e411 assay, Roche Diagnostics, Mannheim, Germany).

Calcium and phosphorus serum levels were closely monitored postoperatively. In case 2 calcium gluconate was administered intravenously, on postoperative day 2, to treat a calcium level drop to 7.2 mg/dl, which was normalised 24 hours later. In all three patients the serum intact PTH (parathormone) levels increased progressively. PTH levels had been restored to normal by the fourth postoperative week, allowing us to wean the patients off calcium and vitamin D_3 _supplementation. No further PTH fluctuations were noted in the patients' follow-up visits over the next 6 months, which was considered indicative of excellent parathyroid graft function [Figure 1]. Moreover, serum calcium and phosphorus levels remained normal throughout the entire 6-month postoperative follow-up period. The patients' preoperative attributes and postoperative course are summarised in Table 1.

**Table 1:** Patients' demographics, diagnosis, management and postoperative course.

	Case 1	Case 2	Case 3
**Age**	65	70	63

**Gender**	Female	Female	Male

**Cause**	Crico-oesophageal carcinoma	Hypopharyngeal carcinoma	Carcinoma of the lower third of the oesophagus and toxic goiter

**Operation**	Transhiatal oesophagolaryngopharyngeal resection and bilateral neck dissection	Transhiatal oesophagolaryngopharyngeal resection and bilateral neck dissection	Transhiatal oesophagectomy and total thyroidectomy

**Postoperative supplementation per day**	Levothyroxine 100 μg, vitamin D_3 _1 μg, and calcium carbonate 1 g	Levothyroxine 100 μg, vitamin D_3 _1 μg, and calcium carbonate 1 g	Levothyroxine 100 μg, vitamin D_3 _1 μg, and calcium carbonate 1 g

**Wean off calcium and vitamin D_3_**	25^th ^postoperative day	28^th ^postoperative day	26^th ^postoperative day

## Discussion

Originally described by Lahey in 1926 [5], parathyroid autotransplantation has been used ever since as a means of preventing postoperative hypoparathyroidism. It can be performed either intraoperatively (immediate autotransplantation), whenever the viability of the parathyroid glands is considered to be unavoidably compromised or in a reasonable time period postoperatively (delayed autotransplanation), when there is evidence that a patient is suffering from permanent hypoparathyroidism [1]. The incidence of hypoparathyroidism following cervical exploration for parathyroid hyperplasia or repeated operations for recurrent or persistent disease has been reported to be as high as 20% [6]. Radical head and neck surgery combined with total thyroidectomy is an indication for parathyroid autotransplantation [7]. Furthermore, aggressive surgical procedures in this anatomical region can also result in hypoparathyroidism due to devascularisation or excision of the parathyroid glands, either incidentally or for adequate oncologic margin achievement.

The aim of our paper is to highlight that parathyroid autotransplantation should be considered as an alternative in all patients subjected to operations in the neck region, involving extensive surgical manipulation or radical resection for oncological reasons, in order to prevent severely disabling permanent hypoparathyroidism. Immediate autotransplantation into the ipsilateral sternocleidomastoid muscle was performed in our first two cases and into brachioradialis muscle of the non-dominant arm in our last case. The pectoralis major is also an unproblematic alternative implantation site, as it is usually included within the operative drape field and well away from the neck region [1]. The muscle pockets used should invariably be re-approximated with either radio-opaque metal clips or long-tailed, non-absorbable monofilament sutures (as performed in our three patients), in order to aid graft localisation in case of a re-operation [8].

Parathyroid gland tissue has been shown to induce angiogenesis spontaneously by increasing the expression of vascular endothelial growth factor, which is the main reason for the feasibility of autograft transplantation [9]. The apparent length of time for vascular in-growth and graft function to develop is approximately 10-20 days following implantation [10]. Our 3 patients displayed normal serum PTH levels and were eventually weaned off calcium and vitamin D_3 _supplementation during their 4^th ^postoperative week. The observation that this time interval was in accordance with the expected time for full graft function reported in the literature, combined with the initial drop of the serum PTH which was noted immediately postoperatively, led to the assumption that permanent hypoparathyroidism was avoided in our patients due to successful autotransplantation. However, the possibility that PTH levels were restored to normal owing to an in situ preserved parathyroid gland that was not identified intraoperatively cannot be fully dismissed.

Apart from immediate autotransplantation, Wells et al [10] described in 1974 the cryopreservation technique according to which the resected parathyroid tissue was placed into a sterile ice bath and 30-40 slivers of it were prepared and transported to laboratory vials containing 10% dimethyl sulfoxide, 10% autologous serum and 80% Waymouth's MB752/1 tissue culture media. These vials were placed in the liquid nitrogen-freezing compartment of a controlled rate freezer and frozen at -1°C per minute to -80°C. Finally, the vials were transported immediately into a liquid nitrogen storage freezer to be maintained at -170°C. It has been stated that the viability of stored parathyroid tissue is greater when the time of cryopreservation does not exceed 24 months [4]. Moreover, Borot et al have reported that 95% of patients who require delayed parathyroid autotransplantation are operated within 24 months following cryopreservation [11]. Therefore, it has been proposed that cryopreservation of the parathyroid glands should not exceed the period of 2 years postoperatively [1].

Highly successful rates of parathyroid graft function have been reported for immediate (85-99% efficacy) versus delayed autotransplanation [6], rendering the first option more favourable. However, in cases where late postoperative hypoparathyroidism is reasonably anticipitated to develop, the resection, cryopreservation and delayed transplantation of parathyroids is a reliable alternative that the surgeon should take under consideration [10,12].

## Conclusions

Although preservation of parathyroid glands in situ is desirable, parathyroid autotransplantation, immediate or delayed, is a simple and safe technique which can be implemented in order to eliminate the risk for postoperative hypoparathyroidism following radical operations of the neck for oncological reasons. However, the potential parathyroid grafts must be pathologically proved not to be invaded by malignant cells before implantation. Considering the relatively rare need to employ parathyroid autotransplantation, it is at the surgeon's courtesy to identify which patients can benefit from using this technique.

## Consent

Written informed consent was obtained from each patient for the publication of this case series report and of any accompanying images. A copy of the written consent is available for review by the Editor-in-Chief of this journal.

## Competing interests

The authors declare that they have no competing interests.

## Authors' contributions

PA participated in the surgical procedures, conceived and designed the study, and wrote the manuscript. MK assisted in drafting the manuscript and revised it for critical content. NA and AM analysed the data and drafted the manuscript. DD participated in the surgical procedures, acquired the data and helped in writing the manuscript. AT helped in the acquisition and interpretation of data. TT and IV revised critically the manuscript. VS carried out the surgical procedures, participated in designing the study and finally revised the manuscript for submission. All authors have read and approved the final manuscript.

## References

[B1] Moffett JM Suliburk J Parathyroid autotransplantation Endocr Pract 2011 17 Suppl 1 83 9 21324813 10.4158/EP10377.RA

[B2] Enzinger PC Mayer RJ Esophageal Cancer N Engl J Med 2003 349 2241 52 10.1056/NEJMra035010 14657432

[B3] Muller JM Erasmi H Stelzner M Zieren U Pichlmaier H Surgical therapy of oesophageal carcinoma Br J Surg 1990 77 845 57 10.1002/bjs.1800770804 2203505

[B4] Guerrero MA Cryopreservation of parathyroid glands Int J Endocrinol 2010 2010 829540 21197072 10.1155/2010/829540 PMC3004380

[B5] Lahey FH The transplantation of parathyroids in partial thyroidectomy Surg Gynecol Obstet 1926 62 508 9

[B6] Feldman AL Sharaf RN Skarulis MC Bartlett DL Libutti SK Weinstein LS Marx SJ Norton JA Fraker DL Alexander HR Results of heterotopic parathyroid autotransplantation: a 13-year experience Surgery 1999 126 1042 8 10.1067/msy.2099.101580 10598186

[B7] Niederle B Roka R Brennan MF The transplantation of parathyroid tissue in man: development, indications, technique, and results Endocr Rev 1982 3 245 79 10.1210/edrv-3-3-245 6749486

[B8] Baumann DS Wells SA Jr Parathyroid autotransplantation Surgery 1993 113 130 3 8430361

[B9] Carter WB Uy K Ward MD Hoying JB Parathyroid-induced angiogenesis is VEGF-dependent Surgery 2000 128 458 64 10.1067/msy.2000.107102 10965318

[B10] Wells SA Jr Farndon JR Dale JK Leight GS Dilley WG Long-term evaluation of patients with primary parathyroid hyperplasia managed by total parathyroidectomy and heterotopic autotransplantation Ann Surg 1980 192 451 8 10.1097/00000658-198010000-00003 7425691 PMC1346985

[B11] Borot S Lapierre V Carnaille B Goudet P Penfornis A Results of cryopreserved parathyroid autografts: a retrospective multicenter study Surgery 2010 147 529 35 10.1016/j.surg.2009.10.010 20153007

[B12] Olson JA Jr DeBenedetti MK Baumann DS Wells SA Jr Parathyroid autotransplantation during thyroidectomy: Results of long-term follow-up Ann Surg 1996 223 472 8 discussion 478-80 10.1097/00000658-199605000-00003 8651738 PMC1235165

